# Overexpression of Heat Shock Protein 70 Ameliorates Meat Quality of Broilers Subjected to Pre-Slaughter Transport at High Ambient Temperatures by Improving Energy Status of Pectoralis Major Muscle and Antioxidant Capacity

**DOI:** 10.3390/antiox11081468

**Published:** 2022-07-27

**Authors:** Tong Xing, Xinglian Xu, Lin Zhang, Feng Gao

**Affiliations:** 1 Key Laboratory of Animal Origin Food Production and Safety Guarantee of Jiangsu Province, Joint International Research Laboratory of Animal Health and Food Safety, Jiangsu Collaborative Innovation Center of Meat Production and Processing, Quality and Safety Control, National Experimental Teaching Demonstration Center of Animal Science, College of Animal Science and Technology, Nanjing Agricultural University, Nanjing 210095, China; zhanglin2012@njau.edu.cn; 2 College of Food Science and Technology, Nanjing Agricultural University, Nanjing 210095, China; xlxus@njau.edu.cn

**Keywords:** heat shock protein, stress, energy metabolism, redox status, meat quality, broiler chicken

## Abstract

The induction of heat shock protein 70 (HSP70) potentially mediates meat-quality development under stress conditions. To investigate the effects and mechanism of HSP70 on the meat quality of the pectoralis major (PM) muscles of broilers exposed to pre-slaughter transport, a total of 168 broilers were intraperitoneally injected with L-glutamine (Gln) or saline. Twenty-four hours later, broilers were subjected to transport or held under normal living conditions. The results indicated that acute Gln supplementation significantly increased HSP70 expression in the PM of transported broilers (*p* < 0.05). The overexpression of HSP70 significantly alleviated the decreases in muscle pH and water-holding capacity and improved the shrinking of muscle fibers induced by transport (*p* < 0.05). HSP70 induction increased ATP content, decreased the activities of glycolytic enzymes, and lowered the phosphorylation level of AMP-activated protein kinase in transported broilers (*p* < 0.05). In addition, the overexpression of HSP70 greatly increased total superoxide dismutase and the total antioxidant capability and decreased the levels of reactive oxygen species, malonaldehyde, and carbonyls in the PM of transported broilers (*p* < 0.05). Overall, this work indicated that HSP70 could effectively improve the meat quality of transported broilers by improving the energy status, inhibiting glycolytic influx, and restoring redox homeostasis.

## 1. Introduction

Transport from farm to abattoir constitutes one of the indispensable pre-slaughter handling procedures of poultry. Various potential stress factors, including the mixing of unfamiliar groups, the stocking density, environmental changes, feed and water deprivation, and road conditions, are involved in the transport process and inevitably lead to physical, psychological, and physiological changes in broiler chickens [1]. Previous studies have evidenced that pre-slaughter transport under conditions of high ambient temperatures can induce severe stress responses in broilers by increasing plasma corticosterone content, and the activities of creatine kinase and lactate dehydrogenase [2,3]. In addition, pre-slaughter transport can cause undesirable changes in broiler meat quality and even increase the incidence of pale, soft, and exudative (PSE)-like meat [4,5]. 

The physiological and metabolic changes that occur upon slaughter play critical roles in the conversion of muscle into meat and the ultimate formation of meat quality [6]. Rapid pH decline, combined with high muscle temperature during the early post mortem period, can induce the denaturation of myofibrillar and sarcoplasmic proteins from muscle, thereby leading to a pale color, decreased water-holding capacity (WHC), and impaired protein functionality during processing [7]. Several studies have demonstrated that pre-slaughter transport impairs the energy status and leads to the activation of AMP-activated protein kinase (AMPK) in the pectoralis major (PM) muscle of broilers, which contributes to the accelerated glycolytic rate and the consequent fast pH decline [2,8]. Poultry meat is particularly vulnerable to oxidative damage due to the high unsaturation levels of lipids and endogenous initiators, including heme iron and certain oxidoreductase enzymes, which reduces the sensory, nutritional, and technological quality of chicken meat products [9]. Pre-slaughter transport has been indicated to induce the overproduction of reactive oxygen species (ROS), thereby leading to oxidative stress and enhancing lipid peroxidation and protein oxidation in the PM muscle of broilers [10,11]. Consequently, strategies aimed at restoring energy homeostasis and redox status have great potential in alleviating pre-slaughter stress-induced detrimental effects and improving chicken-meat quality.

Living organisms have protective measures against various challenges consisting in synthesizing a set of molecular chaperones called heat shock proteins (HSPs). As one of the highly conserved and ubiquitously expressed HSPs, HSP70 has been implicated in maintaining protein homeostasis, alleviating oxidative damage, and regulating apoptosis [12]. A large number of studies have revealed the differential expression of HSP70 in animal muscles following pre-slaughter stress, suggesting its potential roles in mediating the physiological and metabolic events and the development of meat quality [6]. We have recently observed that broilers subjected to acute stress exhibit variations in the expression and distribution of HSP70 in PM muscles and that the insufficiency of HSP70 is correlated with the development of PSE-like meat [13]. In vivo and in vitro studies have evidenced the cytoprotective effects of HSP70 on different chicken tissues and cells under stressful conditions [14,15]. Therefore, we speculate that promoting the induction of HSP70 can improve the resistance to pre-slaughter stress in broilers and alleviate muscle dysfunction. 

L-glutamine (Gln), a conditional essential amino acid, is abundant in skeletal muscle and plays important regulatory roles in improving the cellular redox state, attenuating the inflammatory response, and modulating the synthesis of HSPs [16]. Gu et al. [14] have indicated that the intraperitoneal injection of Gln elevates HSP70 expression in the jejunal mucosa of chickens under acute heat stress, which ameliorates oxidative injury in mucosal tissues and improves intestinal-barrier functions. In addition, oral Gln supplementation induces HSP70 protein expression, thereby attenuating oxidative stress and inflammatory responses and alleviating skeletal-muscle protein degradation in rat models of muscle disuse [17]. Therefore, this study was conducted using Gln to evaluate the effect of HSP70 expression on the meat quality, energy metabolism, and redox status in the PM muscles of broilers subjected to pre-slaughter transport.

## 2. Materials and Methods

### 2.1. Birds and Experimental Design

All experimental procedures and bird management were approved by Nanjing Agricultural University Institutional Animal Care and Use Committee under protocol number SYXK 2016-0054. A total of 250 one-day-old male Arbor Acres broiler chicks were obtained from a commercial hatchery (Ecolovo Poultry Breeding Co., Ltd., Pingyi, China). All birds were provided with commercial standard diets and management during the growth period. At 42 days of age, a total of 168 broiler chickens with similar body weight (BW; 2.52 ± 0.21 kg) were selected and injected intraperitoneally with either Gln (0.75 mg/kg of BW in saline; Sigma-Aldrich Co. Ltd., Shanghai, China) or saline, with an injection dosage of 0.5 mL, according to Hao et al. [18]. Then, birds were labeled and put back into their original cages. Feed and water were provided during the initial 16 h and deprived during the subsequent 8 h after injection. Twenty-four hours later, broilers were held in cages (76 cm × 48 cm × 38 cm) and equally allocated to one of the following four treatments: (1) broilers that received saline injection and were held under normal living conditions (CON); (2) broilers that received saline injection and were subjected to pre-slaughter transport (T); (3) broilers that received Gln injection and were held under normal living conditions (CG); (4) broilers that received Gln injection and were subjected to pre-slaughter transport (TG). Each treatment consisted of 6 replicate cages and 7 broilers per cage. The temperature and relative humidity (RH) inside the chicken house were 25.1 °C and 59.8%, respectively. The transport period lasted for 0.5 h, the average speed of the truck was 45 km/h. During the transport period, the ambient temperature and RH were 31.5–32.7 °C and 68.2–72.3%, and the temperature and RH inside the cages were 37.5 °C and 78.5–82.1%, respectively.

### 2.2. Slaughter and Sample Collection

At the end of the experimental trial, two birds per cage were taken out, electrically stunned (40 V alternating current; 400 Hz for 5 s each), and slaughtered via exsanguination. Within 20 min post mortem, 5 g of PM muscle of the left-wing side was taken, snap-frozen in liquid nitrogen, and then stored at −80 °C until required for further analyses. After scalding and evisceration, the entire right PM muscles were cut from the carcasses and stored at 4 °C for meat-quality determination.

### 2.3. Meat-Quality Measurements

Muscle pH values 0.5 h and 24 h post mortem were measured by inserting a glass-probe electrode into the cranial part of PM muscle using a portable electronic pH meter (Hanna Instrument Company, Porto, Portugal). Meat color including lightness (L*), redness (a*), and yellowness (b*) 24 h post mortem was measured in triplicate at the cranial section of the dorsal surface using a CR400 chromameter (Konica Minolta Company, Tokyo, Japan). Drip loss was assessed according to a previous study with slight modifications [10]. Briefly, duplicate meat samples were trimmed into 1.5 cm × 1.5 cm × 4 cm strips and suspended on a hook in an air-tight container at 4 °C for 24 h. Any moisture on the meat surface was dried using filter paper. Drip loss was measured by calculating the percentage of weight loss during storage. For cooking-loss assessment, PM muscle samples (approximately 50 g) with similar sizes and thicknesses were placed in polyethylene bags and then cooked in a water bath at 80 °C until the internal temperature reached 72 °C. After cooking, the samples were cooled to room temperature and weighed to calculate the cooking-loss percentage after being patted dry.

### 2.4. Hematoxylin and Eosin Staining and Microscopic Analysis

Twenty-four hours post mortem, PM muscle tissues (0.3 cm × 0.3 cm × 1 cm) were cut along the direction of muscle fibers, fixed in freshly prepared 4% paraformaldehyde (pH 7.0), processed, and embedded in paraffin for sectioning and staining. The embedded muscle tissues were sectioned at a thickness of 6 μm and subjected to hematoxylin and eosin (H&E) staining [19]. Images were captured using a Carl Zeiss Axio Scope.A1 light microscope (Oberbochen, Germany). Four fields per section and three sections from each sample were acquired. For quantification, the diameters of the muscle fibers were measured using Image J software, and the average was used for the calculation. Muscle-fiber frequency was analyzed according to Gomez-Perez et al. [20].

### 2.5. Muscle-Lactate, -Glycogen, and -Glycolytic-Potential Determination

Muscle lactate content was determined using a commercial assay kit (Nanjing Jiancheng Bioengineering Institute) according to the manufacturer’s instructions. Muscle glycogen content was measured using the method by Hambrecht et al. [21] with slight modifications. Briefly, 1 g of muscle sample was homogenized in 9 mL of chilled perchloric acid solution (0.85 M HClO_4_) and centrifuged at 2000× *g* for 10 min at 4 °C. The supernatant was collected and neutralized with KOH (10 M). Glycogen in the supernatant fraction was mixed with amyloglucosidase (Aladdin, Shanghai, China) in sodium acetate buffer (0.4 M; pH 4.8) and incubated at 55 °C for 2 h to be hydrolyzed to glucose. Glucose was further determined using a commercial glucose oxidase kit (Solarbio Life Science, Beijing, China). Using this method, the concentrations of glucose-6-phosphate and glucose were not individually determined but included in the glycogen determination. Finally, the glycolytic potential (GP) was calculated as GP = 2 × [glycogen] + [lactic acid] and expressed as μmol of lactic acid equivalent per gram of fresh muscle [22]. 

### 2.6. Muscle-Adenosine-Phosphate Assessment

The concentrations of adenosine phosphates, including adenosine triphosphate (ATP) and adenosine monophosphate (AMP), in PM muscle were determined with high-performance liquid chromatography (HPLC) as previously described [8]. Briefly, 0.5 g of frozen sample was homogenized in 3 mL of ice-cold HClO_4_ (7%) and centrifuged at 15,000× *g* for 10 min at 4 °C. The supernatant was neutralized with KOH (0.85M) and centrifuged again at 15,000× *g* for 10 min at 4 °C to remove KClO_4_. The neutralized supernatant was then passed through a 0.22 μm filter before injection into the HPLC (Waters Corporation, Milford, MA, USA). The chromatographic parameters were set as follows: mobile phase flow rate, 1.0 mL/min; ultraviolet detection wavelength, 254 nm; mobile phases, a mixture of phosphate buffer and methanol (86.5:13.5). Peaks were identified and quantified by comparing the retention time and the peak area with known external standards purchased from Sigma-Aldrich (St Louis, MO, USA).

### 2.7. Muscle-Redox-Status Analysis

Muscle samples were homogenized in ice-cold saline (1:9 (*w/v*)) and centrifuged at 3000× *g* for 10 min at 4 °C. The supernatants were collected and stored at −20 °C for the measurements of the total antioxidant capacity (T-AOC), superoxide dismutase (SOD), glutathione peroxidase (GSH-Px), protein carbonyl, and malondialdehyde (MDA), which were determined using commercial kits obtained from Nanjing Jiancheng Bioengineering Institute (Nanjing, China). The protein concentrations of the supernatants were determined using the bicinchoninic acid (BCA) protein assay kit (Thermo, RD, USA).

The intracellular reactive oxygen species (ROS) level was measured using a dichlorofluorescein probe (Nanjing Jiancheng Bioengineering Institute, Nanjing, China) according to a previous study [10]. The fluorescence intensity was detected at an excitation wavelength of 488 nm and emission wavelength of 525 nm using fluorescence Microplate Reader (Spectramax, MD, USA).

### 2.8. Activities of Muscle Glycolytic Enzymes

Muscle tissues were homogenized in ice-cold saline and centrifuged at 3500× *g* for 10 min at 4 °C. The supernatants were collected and used for the determination of the activities of hexokinase (HK), pyruvate kinase (PK), and lactate dehydrogenase (LDH) using commercial kits (Nanjing Jiancheng Biochemical Institute, Nanjing, China). The protein concentrations of the supernatants were determined using the BCA protein assay kit.

### 2.9. Total Protein Extraction and Immunoblotting Analysis

Muscle samples were homogenized in RIPA lysis buffer (Beyotime Biotechnology, Shanghai, China) containing protease and phosphatase inhibitor mixture (Roche Applied Science, Indianapolis, IN, USA). The homogenates were centrifuged at 12,000× *g* for 20 min at 4 °C. The supernatants were collected, and the BCA protein assay kit was used to determine the protein concentrations. Appropriate amounts of protein were dissolved in 2 × loading buffer (10-mM Tris-HCl, 2.5% SDS, 1% β-mercaptoethanol, 10% glycerol, and 0.01% bromophenol blue; pH 6.8) and heated for 5 min at 95 °C. The immunoblotting analyses were performed as previously described [3]. Primary antibodies against AMPK and phosphor-AMPK (Thr172) were purchased from Cell Signaling (Beberly, MA, USA). HSP70 and glyceraldehyde-3-phosphate dehydrogenase (GAPDH) antibodies were purchased from Bioworld Technology (Nanjing, China). The density of bands was quantified and normalized to the GAPDH content.

### 2.10. RNA Extraction, cDNA Synthesis, and Quantitative Real-Time PCR

Total RNA extraction was performed using Trizol reagent (Takara Biotechnology Co., Ltd., Dalian, China) following the manufacturer’s protocol. cDNA was reverse-transcribed using a commercial kit, cDNA Synthesis Kit (PrimeScript™ RT Master Mix; Takara, San Jose, CA, USA). Quantitative Real-Time PCR was performed using SYBR Premix EX Taq (Takara, USA) on an Applied Biosystems 7500 instrument (Foster City, CA, USA) as previously described [13]. The primer sequences used for quantitative RT-PCR analyses were as follows: HSP70 (forward, 5′-GGAGATCATTGCCAACGACC-3′; reverse, 5′-TTGTACTCCACCTGCACCTT-3′); GAPDH (forward, 5′-CTTTGGCATTGTGGAGGGTC-3′; reverse, 5′-ATGACTTTCCCCACAGCCTT-3′). HSP70 gene expression was calculated as the relative fold changes compared with CON, and GAPDH was used as the internal reference to normalize the expression of target genes. Relative mRNA expression was calculated with the 2^−ΔΔCT^ method.

### 2.11. Statistical Analysis

The data were analyzed with the cage as the experimental unit (*n* = 6; the means of two birds per cage were used to represent the cages). Statistical analyses were performed using one-way analyses of variance (ANOVAs) followed by Duncan’s multiple-range tests in SAS 9.12 (SAS Inst. Inc., Cary, NC, USA). Data were reported as means ± standard error (SE). Significance was considered at *p* ≤ 0.05.

## 3. Results

### 3.1. Levels of HSP70

**Figure 1:**
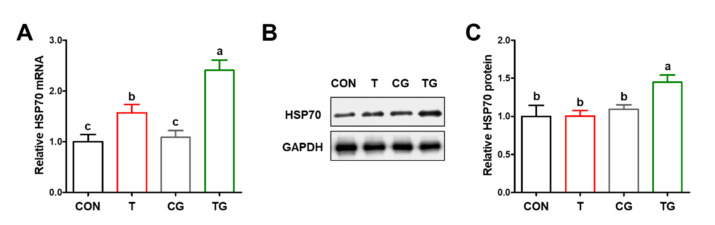
Effect of intraperitoneal injection of glutamine on levels of heat shock protein 70 (HSP70) in pectoralis major muscles of broiler chickens subjected to pre-slaughter transport. (**A**) Relative mRNA expression of HSP70. (**B**,**C**) Relative protein content of HSP70. (**C**) CON, broilers received intraperitoneal injection of saline and were held under a normal rearing condition; T, broilers that received intraperitoneal injection of saline and were subjected to pre-slaughter transport treatment; CG, broilers that received intraperitoneal injection of glutamine and were held under a normal rearing condition; TG, broilers that received intraperitoneal injection of glutamine and were subjected to pre-slaughter transport treatment. All measurements are expressed as means ± SE (*n* = 6) with different superscript letters (a–b) indicating significance (*p* < 0.05).

Compared with the CON group, the mRNA expression of HSP70 was significantly increased (*p* < 0.05) in the PM muscles of broilers in the T and TG groups (Figure 1A), whereas the protein content of HSP70 was only increased (*p* < 0.05) in the TG group (Figure 1B,C). No significant differences (*p* > 0.05) in mRNA or protein of HSP70 were observed between the CON and CG groups.

### 3.2. Meat-Quality Traits

The effects of HSP70 expression on the meat-quality traits of the PM muscles of broilers in response to pre-slaughter transport are exhibited in Table 1. Broilers in the T and TG groups exhibited significantly higher (*p* < 0.05) L* values, cooking loss, and drip loss and lower (*p* < 0.05) pH_0.5h_ and pH_24h_ than those in the CON group. Broilers in the TG group showed lower (*p* < 0.05) L* values and higher (*p* < 0.05) pH_0.5h_, pH_24h_, and water-holding capacity than those in the T group. No significant differences (*p* > 0.05) in meat-quality traits were observed between the CON and CG groups. Moreover, there were no significant differences (*p* > 0.05) in the a* and b* values among the four groups.

### 3.3. Morphological Analysis

**Figure 2:**
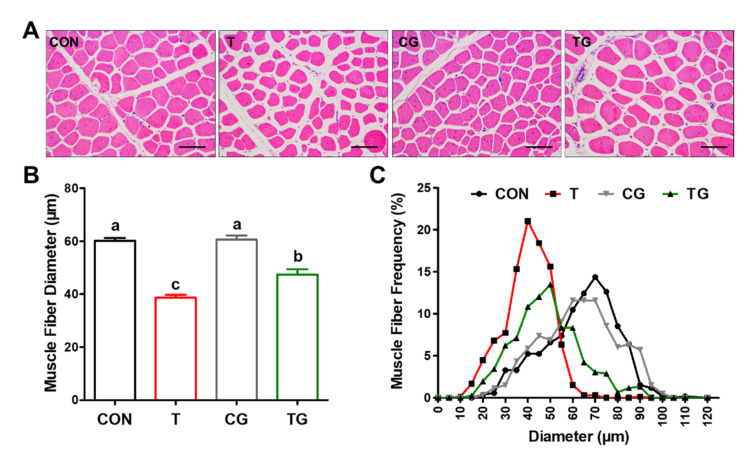
Effects of heat shock protein 70 expression on morphology of pectoralis major muscle fibers of broiler chickens subjected to pre-slaughter transport. (**A**) Representative images of hematoxylin and eosin staining of muscle fibers. (**B**) Diameter of muscle fibers. (**C**) Muscle-fiber distribution. CON, broilers that received intraperitoneal injection of saline and were held under a normal rearing condition; T, broilers that received intraperitoneal injection of saline and were subjected to pre-slaughter transport treatment; CG, broilers that received intraperitoneal injection of glutamine and were held under a normal rearing condition; TG, broilers that received intraperitoneal injection of glutamine and were subjected to pre-slaughter transport treatment. All measurements are expressed as means ± SE (*n* = 6) with different superscript letters (a–c) indicating significance (*p* < 0.05).

H&E staining indicated that transport at a high ambient temperature induced the shrinkage of PM muscle fibers and the increase in endomysial and perimysial spaces (Figure 2A). The average size of muscle fibers was significantly decreased (*p* < 0.05) in transported broilers compared with the CON group (Figure 2B). The muscle-fiber frequency distribution also showed a change towards smaller muscle fibers in the T and TG treatment groups (Figure 2C). The intraperitoneal injection of Gln affected the muscle-fiber size distribution, exhibiting a larger number of bigger muscle fibers and a smaller number of smaller cells (Figure 2A,C). Therefore, the average diameter of muscle fibers significantly increased (*p* < 0.05) after the induction of HSP70 by the injection of Gln in transported broilers (Figure 2B).

### 3.4. Glycolytic Metabolism

**Figure 3:**
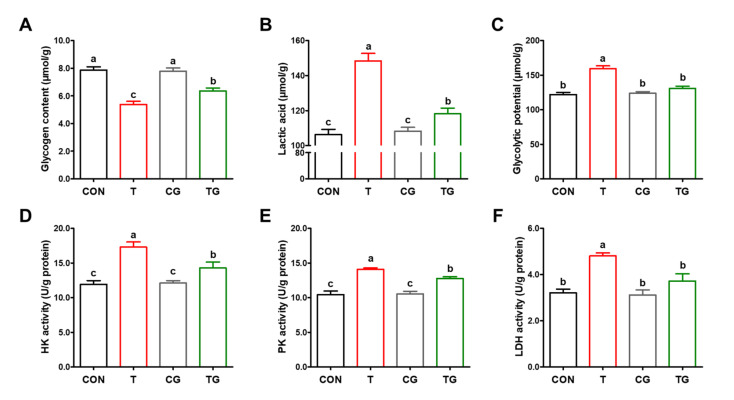
Effects of heat shock protein 70 expression on glycolytic parameters and activities of glycolytic enzymes in pectoralis major muscles of broiler chickens subjected to pre-slaughter transport. (**A**) Glycogen content. (**B**) Lactic acid concentration. (**C**) Glycolytic potential. (**D**) Hexokinase activity. (**E**) Pyruvate kinase activity. (**F**) Lactate dehydrogenase activity. (**F**). CON, broilers that received intraperitoneal injection of saline and were held under a normal rearing condition; T, broilers that received intraperitoneal injection of saline and were subjected to pre-slaughter transport treatment; CG, broilers that received intraperitoneal injection of glutamine and were held under a normal rearing condition; TG, broilers that received intraperitoneal injection of glutamine and were subjected to pre-slaughter transport treatment. All measurements are expressed as means ± SE (*n* = 6) with different superscript letters (a–c) indicating significance (*p* < 0.05).

Compared with the CON group, T treatment significantly decreased (*p* < 0.05) the concentration of glycogen (Figure 3A) and increased (*p* < 0.05) the concentrations of lactic acid and GP in the PM muscles of broilers (Figure 3B). In addition, broilers in the T group had higher (*p* < 0.05) activities of HK, PK, and LDH than those in the CON and CG groups (Figure 3D–F). TG treatment restored the concentration of muscle glycogen and decreased (*p* < 0.05) the concentrations of lactic acid and GP compared with the T group (Figure 3A–C). In addition, broilers in the TG group had significantly lower (*p* < 0.05) activities of HK, PK, and LDH in comparison with those in the T group (Figure 3D–F). Furthermore, there were no significant differences (*p* > 0.05) in the concentration of GP and the activity of LDH between the CON and TG groups.

### 3.5. Energy Status and Activation of AMPK

**Figure 4:**
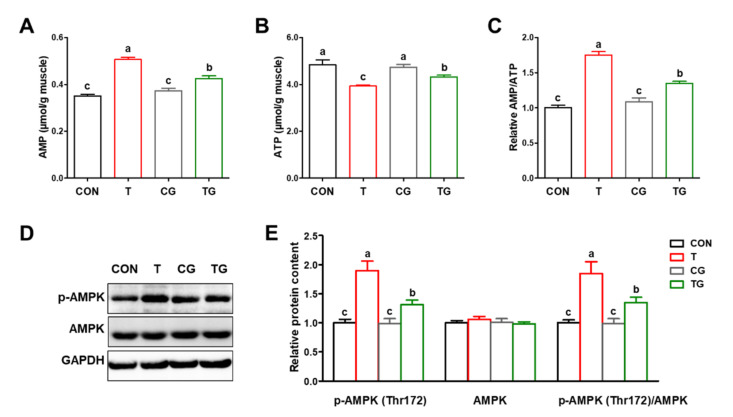
Effects of heat shock protein 70 expression on energy status and activation of AMPK in pectoralis major muscles of broiler chickens subjected to pre-slaughter transport. (**A**) AMP content. (**B**) ATP content. (**C**) AMP/ATP ratio. (**D**,**E**) Phosphorylated/total AMPK contents. CON, broilers that received intraperitoneal injection of saline and were held under a normal rearing condition; T, broilers that received intraperitoneal injection of saline and were subjected to pre-slaughter transport treatment; CG, broilers that received intraperitoneal injection of glutamine and were held under a normal rearing condition; TG, broilers that received intraperitoneal injection of glutamine and were subjected to pre-slaughter transport treatment. All measurements are expressed as means ± SE (*n* = 6) with different superscript letters (a–c) indicating significance (*p* < 0.05).

As shown in Figure 4A–C, broilers in the T group exhibited significantly higher (*p* < 0.05) AMP content and AMP/ATP ratio and lower (*p* < 0.05) ATP content than those in the CON group. The intraperitoneal injection of Gln significantly increased the ATP content and decreased (*p* < 0.05) the AMP content and the AMP/ATP ratio in the PM muscles of transported broilers. In addition, the phosphorylation of AMPK at Thr172 was significantly increased (*p* < 0.05) in the PM muscles of the T group compared with the CON group. In addition, the expression of p-AMPK was significantly downregulated (*p* < 0.05) by the intraperitoneal injection of Gln in transported broilers (Figure 4D,E), indicating that the overexpression of HSP70 restored the energy status and inhibited the activation of AMPK signaling. 

### 3.6. ROS Generation and Redox Status

As shown in Table 2, broilers in the T group exhibited higher (*p* < 0.05) levels of ROS, MDA, and carbonyls than those in the CON group. Broilers in the TG group showed higher (*p* < 0.05) levels of ROS and MDA, as well as enhanced (*p* < 0.05) activities of T-AOC, T-SOD, and GSH-Px, than those in the CON group. In addition, broilers in the TG group had significantly lower (*p* < 0.05) levels of ROS, MDA, and carbonyls and higher (*p* < 0.05) activities of T-AOC and T-SOD than those in the T group. There were no significant differences (*p* > 0.05) in the ROS level or redox status between the CON and CG groups.

## 4. Discussion

Severe acute stress due to the improper pre-slaughter handling and slaughter procedure of poultry has been reported to induce serious welfare and meat-quality problems in broilers, such as morbidity and mortality, as well as the increased occurrence of PSE-like meat [6,23]. When living organisms encounter a variety of stimuli, a group of highly conserved and ubiquitously expressed proteins named HSPs are synthesized and play critical roles in the cellular defense system. As one of the most important members of the HSP family, HSP70 mediates a wide range of cellular activities, including maintaining protein homeostasis, alleviating oxidative damage, and regulating energy metabolism [12]. A large number of studies have demonstrated that HSP70 can be induced upon thermal challenge and that the expression of HSP70 is linked with the morphological changes in the intestine and myocardium of broiler chickens [18,24]. In addition, Hao et al. [25] have observed a significant increase in HSP70 protein content in the PM muscles of broilers subjected to 2 h heat-exposure stress at 40 °C, which is not consistent with the present study. The current study indicated that the induction of HSP70 in the PM muscles of broilers experiencing pre-slaughter transport at high ambient temperature only occurred at the transcriptional level, not at the translational level. This discrepancy suggests that the induction of HSP70 in broilers might be tissue specific and related to the duration or intensity of acute stress. Moreover, Neufer et al. [26] have suggested that the stimulation of HSP70 in skeletal muscle proceeds from type I/IIa to type IIb fibers. As the PM muscle of broiler chickens is composed of almost all type IIb fibers [27], this might also contribute to the relatively slow response of HSP70 induction in the present study. 

Gln has been well documented to enhance HSP70 expression in in vitro and in vivo settings via the activation of the hexosamine biosynthetic pathway, thereby exerting protective effects against cell damage [28,29]. Therefore, we conducted an in vivo HSP70-overexpression chicken model using Gln. The current data indicated that pretreatment with an intraperitoneal injection of Gln significantly increased the mRNA expression and protein content of HSP70 in the PM muscles of broilers after exposure to pre-slaughter transport at high ambient temperatures. This is in accordance with Gu et al. [14], who have indicated that the intraperitoneal injection of Gln induces HSP70 overexpression in the jejunal mucosa of broiler chickens under acute heat stress. Our previous study has demonstrated that the protein content of HSP70 is significantly decreased in the acute stress-induced PSE-like muscles of broilers and has reported high correlations between HSP70, and meat quality and stress indicators [13], suggesting a potential role of HSP70 in chicken-meat-quality development. Herein, Gln-promoted HSP70 significantly alleviated the negative impacts of pre-slaughter transport on the microstructure of muscle fibers and meat-quality traits. 

Exsanguination during the slaughtering process eliminates the oxygen supply and forces metabolically active muscles to switch from aerobic to anaerobic metabolisms to help to meet the energy demand. Post mortem anaerobic glycolysis is largely governed by the degradation of glycogen to lactate and hydrogen ions, which causes the decline in muscle pH. Fast pH decline coupled with relatively high carcass temperatures induces the denaturation of water-binding proteins and expels myowater, which results in the shrinking of muscle-fiber diameters with the enlargement of endomysial and perimysial spaces, the increase in water loss and pale appearance, and even a PSE condition [30]. England et al. [31] indicated that the pH decline rate, especially during the early post mortem period, is closely related to muscle energy availability and demand immediately before slaughter. 

Considerable studies have evidenced that acute heat stress, improper pre-slaughter handling, shackling, and struggle during slaughter can induce a lower energy status in chicken muscle and compel the acceleration of anaerobic glycolysis to maintain energy homeostasis, which subsequently leads to fast pH decline during the early post mortem period and ultimately deteriorates meat quality [8,32,33]. In accordance with these studies, the current results indicated that the pre-slaughter transport of broilers at a high ambient temperature induced exhausted ATP and enhanced the breakdown of glycogen by increasing the activities of HK, PK, and LDH, which led to the accumulation of lactic acid, lower muscle pH, and subsequent poor meat quality. As a central regulator of cellular energy metabolism, the phosphorylation of AMPK was greatly enhanced in the PM muscles of transported broilers, which indirectly increased flux through glycolysis by activating phosphorylase kinase and phosphofructokinase-2 involved in the glycolytic pathway [34]. In spite of this, the increased expression of HSP70 induced by acute Gln pretreatment increased the ATP content, decreased the activities of HK, PK, and LDH, and lowered the phosphorylation level of AMPK in the PM muscles of transported broilers. Stressful preconditioning and HSP70 overexpression have been described to increase its molecular chaperone activity by stabilizing protein molecules under stress conditions in vitro and in vivo. Kabakov et al. [35] have indicated that intracellular-HSP70-level elevation can diminish the cellular ATP-depletion-induced protein aggregation and promote the tolerance to an acute phase of energy-depriving stress. In addition, HSP70 has been demonstrated to prevent electron leaks between complexes III and IV by binding mitochondrial proteins and consequently reducing cytochrome c loss from mitochondrial membranes in response to stress or injury, which contribute to the restoration of mitochondrial respiration and ATP synthesis [36,37]. Our previous study has indicated that HSP70 can interact with several glycolytic enzymes, including phosphofructokinase, pyruvate kinase, and lactate dehydrogenase [38]; the involvement of HSP70 might help to preserve their conformational stability and structural flexibility, thereby maintaining normal glycolytic enzyme activity. Apart from the effect of HSP70, Gln is an important energy substrate for muscle tissue and can also increase cellular ATP production [16]. From the above discussion, it was speculated that the excessive HSP70 induced by the acute supplementation of Gln can effectively restore the muscle energy status, inhibit AMPK activation, and thereby slow down the rapid glycolysis in transport-stressed broilers. 

With the extensive genetic selection toward breast-muscle yield and faster growth rates, modern broiler chickens are particularly susceptible to oxidative stress. Protein oxidation, lipid peroxidation, and the combinations of these modifications can induce conformational and functional modifications of proteins and the formation of protein cross-links, which are considered to be responsible for the undesirable changes in meat quality [9,39]. Multifactorial pre-slaughter stressors have been reported to induce oxidative stress in the skeletal muscle of broilers and cause meat-quality deterioration [6]. Herein, we observed that pre-slaughter transport at high ambient temperature disturbed redox homeostasis and induced oxidative stress in the PM muscles of broilers by increasing ROS generation, lipid peroxidation, and protein oxidation. In addition, the adaptive responses of broilers to pre-slaughter transport stress were insufficient, as the activities of antioxidant enzymes, including T-AOC, T-SOD, and GSH-Px, remained unchanged, which is in accordance with the findings obtained by Liao et al. [40]. HSP70 exerts its cytoprotective effect by facilitating the degradation of damaged oxidized proteins via the ubiquitin-proteasome system, as well as by inducing the expression of key antioxidant enzymes and adjusting their activities under oxidative stress [41]. According to Gu et al. [14], HSP70 significantly elevated antioxidant enzyme activities and inhibited lipid peroxidation to protect the intestinal mucosa of broilers from heat-stress injury. Our results confirmed that the overexpression of HSP70 significantly improved the antioxidant defense system in the PM muscles of broilers during transport, which was reflected by the enhanced activities of T-SOD and T-AOC and the declined levels of MDA and carbonyls. Mitochondria are not only the major source of intracellular ROS but may also suffer from the attack of free radicals [42]. Therefore, the decreased level of ROS in broiler PM muscles due to HSP70 might be related to its protective effect on mitochondrial function.

## 5. Conclusions

In summary, our results indicated that the intraperitoneal injection of Gln enhanced the expression of HSP70 in the skeletal muscles of broiler chickens subjected to pre-slaughter transport at a high ambient temperature. HSP70 induction increased the ATP content, decreased AMP/ATP, and lowered the phosphorylation level of AMPK, which inhibited the activation of glycolytic enzymes in the PM muscles of transported broilers. In addition, the overexpression of HSP70 improved the muscle antioxidant capacity by enhancing the activities of T-SOD and T-AOC, as well as decreasing the ROS level, protein oxidation, and lipid peroxidation. Collectively, the overexpression of HSP70 could ameliorate the acute-stress-induced deterioration of meat quality possibly by improving the energy status and inhibiting glycolytic influx, as well as restoring redox homeostasis.

## Figures and Tables

**Table 1:** Effects of heat shock protein 70 expression on meat-quality traits of pectoralis major muscles of broiler chickens subjected to pre-slaughter transport.

Meat-Quality Traits	Treatments ^1^				SEM	*p*-Value
	CON	T	CG	TG		
L* (lightness)	50.12 ^c^	53.62 ^a^	50.46 ^cb^	51.44 ^b^	0.33	<0.001
a* (redness)	2.87	2.82	3.07	2.92	0.24	0.902
b* (yellowness)	3.72	4.02	3.60	4.30	0.46	0.707
pH_0.5h_	6.48 ^a^	6.30 ^c^	6.45 ^a^	6.38 ^b^	0.02	<0.001
pH_24h_	5.88 ^a^	5.71 ^c^	5.85 ^ba^	5.80 ^b^	0.02	<0.001
Cooking loss (%)	12.60 ^c^	19.17 ^a^	12.79 ^c^	15.45 ^b^	0.49	<0.001
Drip loss (%)	2.68 ^c^	4.28 ^a^	2.82 ^cb^	3.22 ^b^	0.16	<0.001

^1^ CON, broilers that received intraperitoneal injection of saline and were held under a normal rearing condition; T, broilers that received intraperitoneal injection of saline and were subjected to pre-slaughter transport treatment; CG, broilers that received intraperitoneal injection of glutamine and were held under a normal rearing condition; TG, broilers that received intraperitoneal injection of glutamine and were subjected to pre-slaughter transport treatment. ^a–c^ Mean values within a row followed by different lowercase superscript letters were significantly different (*p* < 0.05) among groups. The results are presented as means ± SEM, *n* = 6.

**Table 2:** Effects of heat shock protein 70 expression on reactive-oxygen-species production, antioxidant capacity, and contents of oxidative products in pectoralis major muscles of broiler chickens subjected to pre-slaughter transport.

Items ^2^	Treatments ^1^				SEM	*p*-Value
	CON	T	CG	TG		
ROS level (relative to CON)	1.00 ^c^	1.63 ^a^	1.03 ^c^	1.37 ^b^	0.07	<0.001
MDA (nmol/mg protein)	0.17 ^c^	0.34 ^a^	0.17 ^c^	0.25 ^b^	0.01	<0.001
Carbonyls (nmol/mg protein)	0.43 ^b^	0.77 ^a^	0.44 ^b^	0.58 ^b^	0.05	<0.001
T-AOC (U/mg protein)	0.11 ^b^	0.12 ^b^	0.11 ^b^	0.16 ^a^	0.01	0.003
T-SOD (U/mg protein)	11.86 ^b^	11.89 ^b^	11.84 ^b^	13.12 ^a^	0.35	0.048
GSH-Px (U/mg protein)	6.36 ^b^	7.10 ^ba^	5.86 ^b^	8.06 ^a^	0.50	0.028

^1^ CON, broilers that received intraperitoneal injection of saline and were held under a normal rearing condition; T, broilers that received intraperitoneal injection of saline and were subjected to pre-slaughter transport treatment; CG, broilers that received intraperitoneal injection of glutamine and were held under a normal rearing condition; TG, broilers that received intraperitoneal injection of glutamine and were subjected to pre-slaughter transport treatment. ^2^ ROS, reactive oxygen species; MDA, malondialdehyde; T-AOC, total antioxidant capacity; T-SOD, total superoxide dismutase; GSH-Px, glutathione peroxidase. ^a–c^ Mean values within a row followed by different lowercase superscript letters were significantly different (*p* < 0.05) between groups. The results are presented as means ± SEM, *n* = 6.

## Data Availability

The data are contained within the article.
